# Diffusion Weighted Imaging Super-Resolution Algorithm for Highly Sparse Raw Data Sequences

**DOI:** 10.3390/s23125698

**Published:** 2023-06-19

**Authors:** Krzysztof Malczewski

**Affiliations:** Institute of Information Technology, Warsaw University of Life Sciences, 159 Nowoursynowska, 02776 Warsaw, Poland; krzysztof_malczewski@sggw.edu.pl

**Keywords:** diffusion imaging, magnetic resonance, image enhancement

## Abstract

The utilization of quick compression-sensed magnetic resonance imaging results in an enhancement of diffusion imaging. Wasserstein Generative Adversarial Networks (WGANs) leverage image-based information. The article presents a novel G-guided generative multilevel network, which leverages diffusion weighted imaging (DWI) input data with constrained sampling. The present study aims to investigate two primary concerns pertaining to MRI image reconstruction, namely, image resolution and reconstruction duration. The implementation of simultaneous k-q space sampling has been found to enhance the performance of Rotating Single-Shot Acquisition (RoSA) without necessitating any hardware modifications. Diffusion weighted imaging (DWI) is capable of decreasing the duration of testing by minimizing the amount of input data required. The synchronization of diffusion directions within PROPELLER blades is achieved through the utilization of compressed k-space synchronization. The grids utilized in DW-MRI are represented by minimal-spanning trees. The utilization of conjugate symmetry in sensing and the Partial Fourier approach has been observed to enhance the efficacy of data acquisition as compared to unaltered k-space sampling systems. The image’s sharpness, edge readings, and contrast have been enhanced. These achievements have been certified by numerous metrics including PSNR and TRE. It is desirable to enhance image quality without necessitating any modifications to the hardware.

## 1. Introduction

Diffusion magnetic resonance imaging enables the analysis of brain tissue structure at a significantly smaller scale than what can be achieved through empirical voxel resolution when collecting data on water molecule activity in the brain. The effectiveness of this instrument is restricted by various hindrances such as spatial resolution, acquisition time, and potential distortions caused by motion. Super-Resolution Reconstruction has undergone significant development and rigorous testing over the course of the last twenty years. Notwithstanding the advancements made, there remain numerous unresolved issues. Certain entities are subject to regular simplification. Scientists commonly aim to optimize the inter-frame motion scheme. The commonly employed global motion estimation algorithm, which is defined manually, fails to consider the potential motion trajectories that may arise within sets of frames. The algorithm presented in this study integrates discrete dense displacement sampling into its framework, thereby facilitating the deformable registration of high-resolution images. The efficacy of Super-Resolution Reconstruction (SRR) has been evidenced in various medical imaging modalities, including Functional MRI (fMRI) as cited in references [1,2], as well as Computerized Tomography (CT) and Positron-Emission Tomography (PET), as cited in reference [3].

The persuasive argument put forth by the authors suggests that residual learning techniques have the potential to improve the effectiveness of said algorithms. By employing a modified optimization technique that involves eliminating unnecessary modules from conventional residual networks, additional improvements have been attained [4]. Furthermore, the authors have proposed to augment the magnitude of the model. The aforementioned alteration resulted in a stabilization of the training procedure. The utilization of these algorithms in medical image processing poses a challenge due to the necessity of training sets.

**Figure 1:**
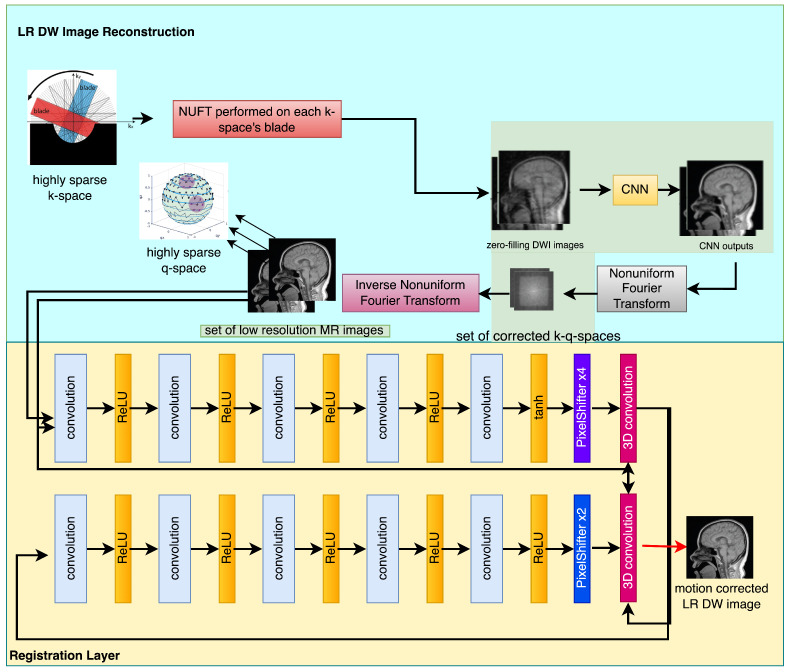
Thesimplified flowchart of the presented low resolution DW image reconstruction from a compressed sensing k-q spaces. Signals are transferred from layer to layer through the input-output manner.

Zhang in [5] provided a technique that may significantly enhance the performance of saliency identification, keypoint recognition, and underwater picture segmentation. The authors of reference [6] employed 2D convolution as a means of extracting both spatial and texture features. Subsequently, an attention mechanism is incorporated into the 2D convolution module to enhance the precision of the spatial and texture characteristics. The utilization of both 3D and 2D convolutions is advantageous due to their complementary properties, which enable the comprehensive exploitation of spatial and textural features in hyperspectral images. In addition, a hyperspectral image dataset comprising 1200 samples of ten distinct corn varieties was developed by them [6]. Empirical investigations conducted on the proposed dataset revealed that SSTNet exhibited superior performance in comparison to the existing cutting-edge techniques for the purpose of identifying corn varieties. Wu et al. (2023) introduced a novel methodology for detecting and quantifying the quantity of banana clusters in intricate orchard settings, during both the SBR and harvesting phases [7]. The authors Zhang et al. conducted an investigation into techniques aimed at enhancing the detection of fruits in intricate surroundings. The authors delineated the prevalent categories of intricate backgrounds observed in open-air orchard settings [8]. The authors Wang et al. (2019) presented a new technique, referred to as HR optical flow estimation (SOF-VSR), which aims to improve the quality of videos. The objective of the shown methodology was to improve the precision of motion compensation through the reconstruction of high-resolution optical flow [9]. The aforementioned result was achieved by approximating optical flow between frames with decreased resolution. Chu et al. [10] introduced the topology of Temporally Coherent Generative Adversarial Networks (GANs). The research utilized optical flow networks and novel loss functions to improve temporal coherence. Furthermore, it has been noted that the proposed methodology enhances the perceptual quality of magnetic resonance (MR) images by reducing feature space losses. The utilization of the deformable motion registration network has been observed to enhance the pragmatic outcomes, albeit at the expense of augmented computational and memory expenditures. The presence of artifacts stemming from inaccurate computation of motion vectors would persist in the restored high-definition frame. The successful attainment of super-resolution reconstruction can be credited to the utilization of deformable registration of low-resolution images. The methodology in question involves the analysis of a series of photographic captures, as opposed to being dependent on a solitary image frame. The diagram depicted in Figure 1 portrays the model framework currently being examined. The framework comprises two discrete elements, specifically the deformable registration module and the reconstruction neural network. The second component is composed of two elements, namely, a generator referred to as G and a discriminator designated as D.

The efficacy of employing a Generative Adversarial Network (GAN) architecture for motion correction is predicated on its established capacity to tackle concerns associated with image restoration and facilitate Magnetic Resonance Imaging (MRI) reconstruction. The above-mentioned capability is attributed to its proficiency in generating high-quality images, as evidenced by the reference [11]. The GAN framework consists of two fundamental components, namely, a generator represented by the symbol G, and a discriminator represented by the symbol D. The two aforementioned entities are engaged in a competitive rivalry with the aim of surpassing one another. The principal objective of the generator is to generate samples that exhibit a high degree of similarity to authentic data, whereas the discriminator’s primary aim is to effectively distinguish between genuine and synthetic samples.

The primary cause of image deformations in Magnetic Resonance Imaging (MRI) is the in-plane motion of the patient. However, this motion can also be utilized as a significant advantage for super-resolution. The tasks of image registration and motion artifact removal are complex and are elaborated upon in Section 4.1. Nevertheless, a significant drawback of Magnetic Resonance Imaging (MRI) is its protracted examination duration. In addition to these variables, the hastening of Magnetic Resonance Imaging (MRI) data acquisition has been examined by numerous scholars. One prospective avenue for future development involves modifications to the phase encoding intervals utilized during k-space filling. Regrettably, this attribute commonly leads to a decline in the quality of the image. Thankfully, the suggested k-space sampling pattern presents a viable solution to circumvent this issue. The utilization of single-shot echo planar imaging (SS-EPI) has gained significant popularity as an acquisition technique in the realm of Diffusion Weighted Imaging (DWI). However, despite its unquestionable importance, specific attributes have persisted as a cause for perplexity and logical discourse. The primary constraint of the imaging technique under consideration is its spatial resolution, which poses challenges in the visualization of small anatomical features. Furthermore, despite the latest advancements in parallelizing input data capture, this obstacle persists as a significant challenge. Cheryauka and colleagues have referenced six suggested techniques for shortening the examination time in multishot diffusion weighted imaging (DWI) [12]. These techniques include capturing only a subset of fast spin echo (FSE) blades in each scan and utilizing a tensor model-based reconstruction. In the late 1990s, the PROPELLER (Periodically Rotated Overlapping ParallEL Lines with Enhanced Reconstruction) procedure was developed by Pipe with the aim of mitigating motion artifacts. The fundamental principle of this approach involves the utilization of a series of radially oriented segments or “blades” to sample k-space. The blade is comprised of multiple parallel phase-encoded lines that can be acquired through the utilization of fast spin echo or gradient echo techniques. The identification of a time-efficient acquisition algorithm involves the selection of a subset of PROPELLER FSE blades per DW image and the utilization of a tensor model-based reconstruction. A modified reconstruction component was proposed as a means of accelerating PROPELLER EPI (echo planar imaging) through a comparable approach. This involved capturing only 10 distinct diffusion directions using a single blade from a given sampling pattern for each direction, and subsequently obtaining a diffusion weighted scan at full resolution by utilizing all of the blades. Despite the promising outcomes of reduced data acquisition in various studies [12,13], the feasibility of extending these approaches to accommodate arbitrary diffusion direction schemes comprising more than ten directions is low. Furthermore, the algorithms utilized are capable of processing data from various diffusion directions to produce a single DW scan. Hence, the strong correlation coefficient observed between neighboring DW images is either disregarded or only partially considered in the literature. DW scans demonstrate comparable diffusion contrast when their diffusion directions are proximate within a q-space sphere. To date, the potential of this similarity to expedite the examination process has not been harnessed.

The authors proposed a method for accelerating data acquisition through the use of a rotating single-shot acquisition (RoSA) procedure with model-free reconstruction, based on the observed correlation. The crux of this algorithm involves acquiring a solitary blade for each diffusion direction in a single shot. The RoSA technique utilizes similarities that are apparent in diffusion weighted contrast for the purpose of image reconstruction. Therefore, it is necessary to utilize a subset of diffusion weighted (DW) data that has diffusion directions in proximity to the DW image under examination. It is assumed that DW scans that are adjacent exhibit a significant correlation coefficient. The modification of the highly constrained back-projection (HYPR) reconstruction procedure was undertaken for the purpose of reconstructing the RoSA images. The HYPR method is a standard non-iterative reconstruction technique utilized in subsampled radial acquisition for time-resolved MRI. According to reference [14], this technique addresses one-dimensional temporal domain correlations. RoSA measures correlations in three-dimensional q-space. Parallel imaging via RoSA reduces data collection time. This study uses the PROPELLER sampling strategy with Compressed Sensing and Partial Fourier methods. According to Partial Fourier, the scanned object’s phase changes gradually. SMASH (simultaneous acquisition of spatial harmonics) and SENSE (sensitivity encoding method) use receiver-coil sensitivity previous information to reduce acquisition time. According to reference [15], conjugate symmetry may rebuild the missing k-space section [16]. Acceleration has been problematic in previous research. Thus, different sample trajectories - rosette, spiral, radial, PROPELLER, and others—have been verified [17,18]. Formally, acceleration efficiency is proportional to receiver coil count. Upon taking into account all hardware constraints, such as noise, the realized level of acceleration is generally reduced. The acceleration rates are generally moderate. The utilization of Compressed Sensing (CS) techniques, as referenced in sources [19,20,21], has the potential to enhance the speed of data acquisition in Magnetic Resonance (MR) imaging. The suitability of MRI as an application of computer science is attributable to its inherent characteristics.The relative ICI (RICI) method is used to create an adaptive data-driven picture denoising technique by [22]. An adaptive image technique for noise reduction enhances X-ray pictures in [23]. The authors of [22,23], in contrast to the presented algorithm, focus on denoising methods while ignoring the proposed algorithm’s features, such as resolution enhancement, compressed sensing, and motion correction. In addition, the proposed method is intended for DW-MRI.

In the meantime, RoSA has demonstrated its effectiveness by permitting high acceleration rates, namely [17]. In this paper, the presented algorithm makes use of its assets, but unlike other algorithms, the input sequences are additionally compressed. The method uses compressed sensing, partial Fourier, super-resolution, GRAPPA, and RoSA. Unlike PI (Parallel Imaging) methods, they may speed up the procedure. Multiple compression-sensing methods have been tested. Due to the differences across k-space sampling methods, several attempts have been made to combine unique, redundant information from different schemes. The CS framework lowers picture noise better than parallel imaging approaches alone.

DW image similarities are ignored by several approaches, including [24,25,26,27]. Despite comparable diffusion disparities, the writers of ref. [28] do not disregard them; q-space has near diffusion directions. The authors claimed their strategy would speed up testing. This work presents a super-resolution, motion correction, and robust sampling trajectory pattern MRI area-associated approach. Experimental findings are promising and demonstrate the method’s utility. The goal was to increase picture resolution and edge delineation while reducing collection time. Thus, the compressed sensing approach may apply irregular sampling processes to well-known PROPELLER blades, benefiting the novel sampling pattern [29]. Like the PROPELLER blades, this k-space filling procedure acquires k-space subsets in one phase encoding direction, but it has been “halved” in Hermitian symmetry of the complex space [30,31]. This advantage makes recovering the missing k-space component easy. This approach improves high-frequency component interpretation.

The main contributions of this work are:

1. The framework algorithm exhibits comprehensiveness with respect to the reconstruction procedure of DW images, sampling schemes of raw data, synchronization of k-q spaces, deblurring, denoising, motion estimation, and ultimately, the reconstruction of super-resolution images.
2. A new GAN-based super-resolution image reconstruction model for DW-MR images is presented.
3. The algorithm extracts image features at different scales. Other authors usually simplify this issue.
4. At various scales, the algorithm extracts visual characteristics. This topic is often simplified by other authors.
5. The technique uses different preprocessing phases for deblurring and denoising layers.
6. The method uses a DW image reconstruction approach based on convolutional neural networks to rebuild low quality images from very sparse k-q spaces.
7. The scheme uses the compressed sensing architecture to focus on reducing acquisition times.
8. The procedure nests the author’s deformable motion estimation procedure as its reconstruction layer.

## 2. The Efficiency of MRI k-Space in Relation to Sparse Sampling Techniques

The utilization of algorithms in compressed sensing requires the acquisition of samples that are entirely random. Subsampling at a rate lower than the Nyquist frequency can lead to the occurrence of aliasing artifacts, thereby necessitating the need to prevent such an occurrence. The model mentioned previously, as referenced in [21], facilitates conduct that is similar to that of additive white Gaussian noise (AWGN). The effectiveness of utilizing a subsampling pattern that is founded on a Gaussian probability density function to acquire samples that are both completely random and incoherent with aliasing was demonstrated through an illustrative example. The acquisition of a truly random subsample can pose difficulties owing to apparent constraints in hardware. The present matter can be partially alleviated by employing matched k-space sampling patterns, which ensure the incoherence of interference and pseudorandomness of the samples (refer to source [23]). According to the paper [27] the implementation of a non-uniform sampling pattern with a linear distribution of power across high-frequency components can lead to advantageous results and mitigate the incidence of aliasing artifacts.

The Poisson-disc sampling methodology generates a dense arrangement of points that are spaced apart by a predetermined minimum distance, resulting in a more naturalistic configuration. This methodology satisfies all necessary requirements, including the absence of coherence and the specified spacing among specimens. Moreover, it ensures the requisite noise characteristics. The concept of sampling low-frequency components at a higher sampling frequency is proposed by the author, as these components are considered to possess significant information.

According to Otazo et al. [19], it is recommended to use a higher sampling frequency for low frequencies as they offer valuable contrast. Their objective is to accelerate the phase contrast cine MRI procedure by combining compressed sensing and parallel imaging techniques. The phrase “k-t SPARSE-SENSE” is being alluded to. The author’s utilization of an altered sampling methodology elicits reflection on the possibility of nonconformity. The k-space sampling algorithm that has been proposed involves the subsampling of PROPELLER blades along the phase-encoding directions, in accordance with a Poisson point process.

Theoretical enhancement of temporal efficiency is the aim of the integration of the Partial Fourier concept with compressively-sensed data, as proposed in [32]. The optimization of raw data redundancy, as elaborated in [13], results in a reduction of scanning times. Considerable resources are dedicated to retrospectively acquiring all samples, which prevents the application of Partial Fourier and Compressed Sensing methodologies. This approach is frequently utilized to specifically capture the high-frequency components. The potential result may entail a reduction in the accuracy of edge demarcation [33]. The aforementioned characteristics of images may have a significant impact on enabling an accurate medical diagnosis. Accurate identification of organ boundaries is of utmost importance in medical contexts, as it facilitates precise diagnoses through the alignment of motion parameters or internal organ characteristics. The presented sampling technique involves an augmented sampling density for high-frequency components, which utilizes Partial Fourier [18] and Compressed Sensing [34]. The utilization of technology enables the achievement of efficient scanning durations while maintaining optimal levels of all essential image attributes. The current investigation presents a sampling methodology that endeavors to minimize redundancy to the maximum degree feasible. The utilization of compressed sensing techniques is a prevalent practice in practical scenarios, wherein it is employed to diminish the dimensionality of subspace sampling trajectories, thereby leading to sparsity. In the acquisition phase, subsets are gathered, which bears resemblance to the PROPELLER concept as cited in reference [29].

On the premise that the subject transitions among obtained k-q-space structural components, the subsequent subgroup is linked to patient motion that may be rectified and integrated into a super-resolution algorithm as its fundamental feature, as illustrated in Figure 1.

The low-resolution DW image reconstruction algorithm commences by utilizing k-q-spaces that are highly compressed. The non-uniform fast Fourier transform (NUFFT) is utilized due to the presence of irregular sampling schemes. The subsequent phase utilizes the zero-filling (ZF) methodology.

The technique of zero-filling is commonly employed for the purpose of increasing the dimensions of the image matrix along the phase-encoded direction. In the context of 2D imaging, the observed rise in matrix size pertains to the “within-plane” dimension, wherein the pixel count escalates from 256 to 512. The utilization of ZIP in 3D imaging can enhance the perceived resolution in the slab-select direction by employing the “through-plane” approach, resulting in an increase from 64 to 128 slices. The benefits are observed only during the initial doubling of the matrix size, beyond which no further advantage is obtained. While the process of zero-filling does not introduce any additional information to the original raw data, it can enhance the perceived spatial resolution of the resulting image by mitigating the effects of partial volume artifacts. The process of zero filling serves as a means of signal interpolation between adjacent voxels, resulting in a more uniform and less granular visual representation of the image.

The convolutional recurrent neural network architecture is utilized in the proposed algorithm for MR image reconstruction. This approach enables the reconstruction of high quality images from highly undersampled k-space data by jointly exploiting the dependencies of the utilized sequences.

The architecture under consideration incorporates the framework of conventional iterative algorithms, adeptly representing the repetition of the iterative reconstruction phases through the utilization of recurrent hidden connections across said iterations. Furthermore, the acquisition of spatio-temporal dependencies is achieved through the utilization of bidirectional recurrent hidden connections across temporal sequences.

Subsequently, a correction in k space is required. The utilization of the inverse non-fast Fourier transform is aimed at generating a sequence of rectified k spaces. Ultimately, a collection of low-resolution diffusion weighted images is obtained through the utilization of an image reconstruction technique that employs the non-uniform fast Fourier transform (NUFFT) as its fundamental component. This study examines the alterations made to the motion effects in order to facilitate the reconstruction of super-resolution magnetic resonance images. Moreover, the registration algorithm exhibits a high degree of precision and effectively mitigates problems related to in-plane and out-of-plane motion.

## 3. The Algorithms for SR Image Reconstruction

**Figure 2:**
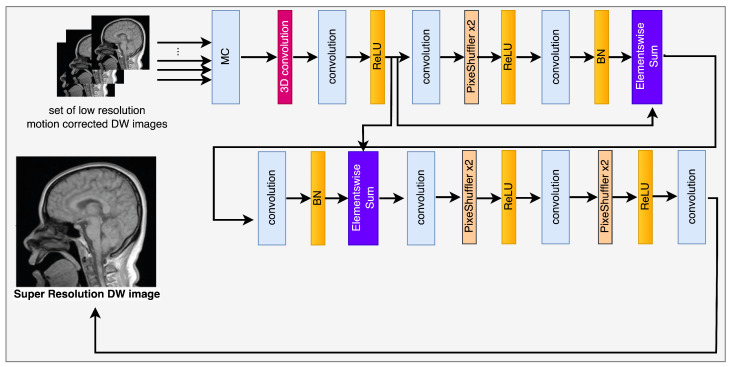
The flowchart of the generator network.

The implementation of multiple frames of images demonstrates temporal correlation between frames, thereby augmenting the information contained within each individual frame. Therefore, it is essential to consider both precision and uniformity simultaneously. Achieving this objective typically involves two primary approaches: motion compensation and super-resolution restoration [35]. The notion of video super-resolution (SR) was initially fragmented into separate subtasks of multi-frame SR, as evidenced by the reference [36]. The main aim was to achieve superior results in image reconstruction for every individual image. However, the high-resolution frames produced independently exhibit a temporal inconsistency issue, leading to the presence of disruptive flickering irregularities. The aforementioned methodologies were insufficient in effectively utilizing the data available in the temporal domain. The integration of optical flow networks in Video Super-Resolution (VSR) with the aim of motion estimation has garnered significant attention in current academic research. Caballero et al. (2017) proposed the utilization of the sub-pixel convolution network model for the purpose of video processing in their research [37]. The approach adopted by the authors was designed to optimize the efficacy of the model under consideration. The current research employed spatio-temporal sub pixel convolution networks and optical flow networks to improve the efficacy of the previously established sub pixel convolutional neural network model [38,39]. Caballero’s model attained expertise in motion compensation through the former and improved its real-time accuracy through the latter. Sajjadi et al. introduced a new methodology termed frame recurrent video super-resolution in their published work [40]. The approach mentioned above utilizes pre-existing SR frames to reconstruct subsequent frames. The methodology being examined in the study has been labeled as “frame and feature-context video super-resolution” by the authors [40]. The methodology involves employing pre-constructed frames with a high level of resolution and reusing features from previous frames in a recurring manner. The authors Wang et al. (2019) proposed a new method, referred to as HR optical flow estimation (SOF-VSR), aimed at enhancing the quality of videos [9]. The objective of the proposed methodology was to improve the precision of motion compensation through the reconstruction of high-resolution optical flow. The aforementioned result was achieved by approximating optical flow between frames with reduced resolution. Chu et al. [10] introduced the topology of Temporally Coherent Generative Adversarial Networks (GANs). The research utilized optical flow networks and novel loss functions to improve the temporal coherence. Furthermore, it has been observed that the proposed methodology has the capability to improve the perceptual quality of Magnetic Resonance (MR) images by reducing feature space losses. The utilization of the deformable motion registration network has been observed to enhance the pragmatic outcomes, albeit at the expense of augmented computational and memory expenditures. The high-definition frame that has been restored may still contain artifacts resulting from inaccurate computation of motion vectors. The successful attainment of super-resolution reconstruction can be credited to the utilization of deformable registration of low-resolution images. The methodology in question involves the analysis of consecutive photographic captures, as opposed to being dependent on a solitary image frame. The diagram depicted in Figure 2 represents the model framework currently being examined. The framework comprises two discrete components, specifically, the deformable registration block and the reconstruction network. The second component is composed of a generator, referred to as G, and a discriminator, designated as D.

The efficacy of employing a Generative Adversarial Network (GAN) architecture for motion correction is predicated on its demonstrated capacity to mitigate challenges associated with image restoration and facilitate Magnetic Resonance Imaging (MRI) reconstruction. The aforementioned capability is attributed to its proficiency in generating high-quality images, as evidenced by the reference [11]. The GAN framework consists of two fundamental components, namely, a generator represented by the symbol G, and a discriminator represented by the symbol D. The two aforementioned entities are engaged in a competitive rivalry with the aim of surpassing each other. The main objective of the generator is to generate samples that exhibit a high degree of similarity to authentic data, whereas the discriminator’s primary goal is to effectively distinguish between genuine and synthetic samples.

(1) $$\underset{G}{min}\underset{D}{max}E_{x}\left[log\left(D(x)\right)\right]-E_{y}\left[log\left(1-G(y)\right)\right].$$

The symbols *y* and *x* represent the graphical representations that have undergone motion distortion and those that have been rectified for it, respectively. The encoder block, excluding the central layer, is composed of five convolutional layers and includes $\frac{n}{2}$ feature maps, with each map containing *n* features. The structural design of the decoder blocks displays resemblances to that of the encoder blocks, with the exception being the utilization of transposed convolutions instead of convolutional layers. The implementation of image registration technology involves the calculation of spatial transformation parameters through an image registration methodology, as described in reference [41]. Following this, the rectification of the incongruity in displacement among consecutive frames is executed. The implementation of displacement parameters facilitates the performance of a spatial transformation on neighboring frames of images, which were obtained at different times and locations, yet depict the identical object.

**Figure 3:**
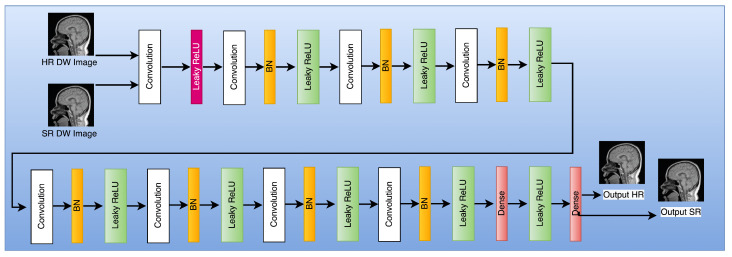
Flowchart of the discriminator network.

The module responsible for registration produces several sets of results, which are subsequently combined with the objective frames denoted by $I_{LR}$ through the utilization of a 3D convolutional layer. The output that is obtained is then fed into the generator network, which is represented as G. The current investigation utilized a generator G-network framework that was based on the SRGAN G-network architecture, as illustrated in Figure 2. The G-network has been developed to integrate a unique residual block with the objective of reducing the parameter count, while preserving the network’s capacity to generalize. The utilization of residual network has been documented in reference [42] to implement two sub-pixel convolutional layers on the image. This approach is adopted to facilitate the achievement of the intended resolution. The discriminator’s architecture, labeled as D in Figure 3, comprises eight convolutional layers in total.

Increasing the number of network layers leads to a corresponding rise in the number of features. The decrease in the number of feature dimensions can be ascribed to the declining magnitude of the convolutional kernel. Two modifications were implemented to address the suboptimal reconstruction outcomes of the SRGAN and to surmount the challenges encountered during the network’s training and convergence. The omission of the Sigmoid activation function from the output layer of the discriminator D was observed in the initial implementation. Additionally, it is noteworthy to mention that the adjustments performed on the parameters were limited to a consistent numerical value denoted as *c* (precisely, 0.01) in terms of absolute magnitude. The study of Generative Adversarial Network (GAN) has gained significant prominence in recent times, owing to its complex and challenging nature [43].The focus of this study is primarily on the insufficiency of security protocols during the training phase and the complex convergence of the model. This is supported by the reference cited as [44]. The observed phenomenon can be explained by the restricted intersection between the authentic and artificially generated distributions. Insufficient attention to the JS divergence, a metric employed for assessing the relative divergence between two probability distributions, may hinder the convergence of the neural network. The study conducted by Arjovsky et al. showcases the effectiveness of the Wasserstein distance in measuring the degree of dissimilarity between two distributions, even when their intersection is minimal.

## 4. The Algorithm for Reconstructing DWI Images with Super Resolution

**Figure 4:**
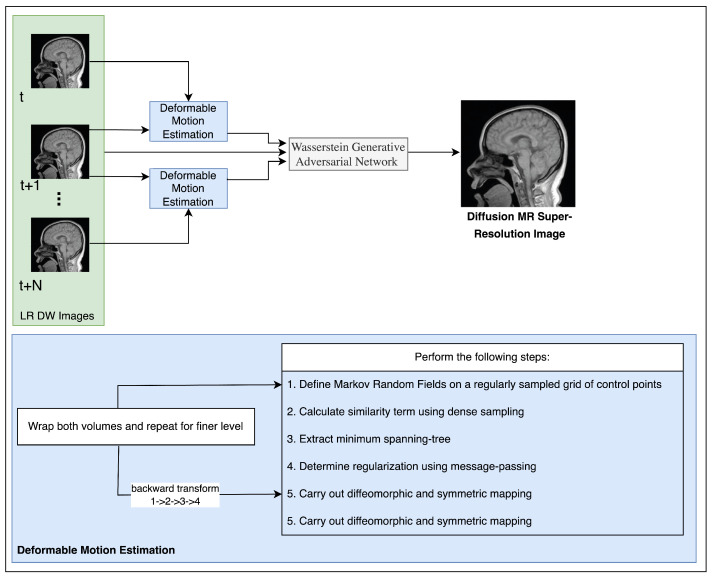
Theproposed DW super-resolution image reconstruction algorithm.

**Figure 5:**
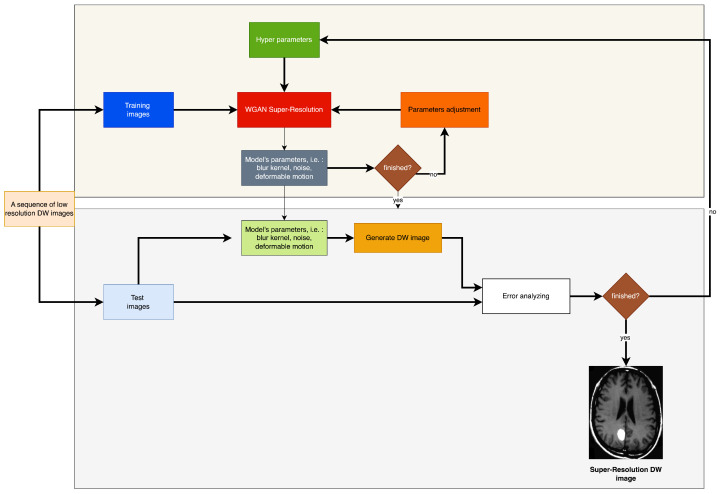
The algorithm utilizes test and train images in order to reconstruct super-resolution DW images.

The algorithm commences by reconstructing a set of low-resolution DWI images from a cluster of highly sparse k-q spaces, as cited in the Figure 1. The algorithm employs deblurring and registration layers, and its primary reconstruction process is structured as depicted in Figure 2, Figure 3, Figure 4 and Figure 5.

The effectiveness of creating a confrontation network through the utilization of the Wasserstein distance is augmented by a particular attribute, as evidenced by the WGAN [45]. The definition of the Wasserstein distance is as follows:

(2) $$W_{d}\left(P_{real},P_{synth}\right)=\frac{1}{K}\underset{{\left||f|\right|}_{L\leq K}}{sub}E_{\left(x,y\right)∼P_{real}}\left[f\left(x\right)\right]-E_{x∼P_{synth}}\left[f\left(x\right)\right].$$

The equation mentioned above employs the mathematical notation $\prod {(P}_{data}),P_{synth}$ to represent the set of all possible joint distributions between $P_{real}$ and $P_{real}$. The equation provided specifies that the symbol $f_{w}$ represents the discrimination function of the adversarial network that was generated. Consequently, the range of the derivative of the input sample for the discriminator is restricted. The discriminator’s parameter *w* is subject to an update process that is restricted to a range of values between −c and *c*. The technique mentioned above is utilized to emphasize the significance of the gradient update generator and mitigate the possibility of the vanishing gradient phenomenon. The function represented by the symbol $f_{w}$ fulfills the following equation:

(3) $$L=E_{x∼P_{real}}\left[f_{W_{d}}\left(x\right)\right]-E_{x∼P_{real}}\left[f_{W_{d}}\left(x\right)\right].$$

With each increment of the variable L, an estimation of the Wasserstein distance between the probability distributions $P_{real}$ and $P_{synth}$ can be obtained. The initial distribution pertains to authentic data, while the subsequent distribution pertains to artificially generated data. The loss functions for the discriminator and generator can be precisely defined as follows:

(4) $$D_{loss}=E_{x∼P_{synth}}\left[f_{W_{d}}\left(x\right)\right]-E_{x∼P_{real}}\left[f_{W_{d}}\left(x\right)\right],$$

(5) $$G_{loss}=E_{x∼P_{synth}}\left[f_{W_{d}}\left(x\right)\right].$$

The function that measures the loss of the discriminator during training, denoted as $D_{loss}$, is a crucial component of the training process. The evaluation of Generative Adversarial Network (GAN) training can be deduced through a decrease in the Wasserstein distance between the authentic and synthesized distributions. This metric exhibits a negative correlation with the magnitude of the distance.

The principal aim of this methodology is to enhance the training process of the generator, denoted as G. The aim of this task is to evaluate the correlation between the input sequence $I_{t}^{LR}\left(t=1,...,N\right)$ and its corresponding counterpart It. The task was accomplished by utilizing a feedforward convolutional nn.

The neural network underwent a training procedure utilizing the parameter identified as $\Psi_{G}$. The literature [36] states that the parameters of a neural network with L layers, denoted as $\Psi_{G}=\left\{W_{1:L};b_{1:L}\right\}$, are acquired through the minimization of the loss function $I_{G}$ for the super-resolution generation network.

(6) $$\Psi_{G}^{*}=\underset{\Psi_{G}}{argmin}\frac{1}{N}\sum_{t=1}^{N}I_{G}\left(G\Psi_{G}I_{t}^{LR},I_{t}^{HR}\right).$$

The present study employs a loss function denoted as $I_{G}$, which is grounded in previous academic literature and has been duly referenced in [36].

(7) $$I_{G}=l_{MSE}+10^{-6}l_{Gen},$$

where $l_{Gen}$ denotes adversarial loss. The SRGAN model integrates the loss functions of the generator and discriminator, denoted as $I_{G}$ and $I_{D}$, respectively, into a comprehensive net loss function. We define $I^{LR}$ by a real-valued tensor of size *W* by *H* by *C* for an image with C color channels, and $I^{HR}$, $I^{SR}$ by rW, rH and *C*, respectively.

(8) $$I_{D}=\frac{1}{N}\sum_{n=1}^{N}\left(log\left(1-D_{\Psi_{D}}\left(G\Psi_{G}I^{SR}\right)\right)\right)-log\left(D_{\Psi_{D}}\left(I^{HR}\right)\right).$$

The provided equation concerns the reconstruction of the generator of the discriminator, which is symbolized as $G\Psi_{G}I^{SR}$, and the initial image $I^{HR}$, which is denoted by $D_{\Psi_{D}}\left(G\Psi_{G}I^{SR}\right)$ and $D_{\Psi_{D}}\left(I^{HR}\right)$, correspondingly. The symbol *N* denotes the quantity of target images. The symbols $I_{G}$, $l_{MSE}$, and $l_{Gen}$ denote variables that have been explicitly defined in the following manner:

(9) $$l_{MSE}=\frac{1}{r^{2}HW}\sum_{x=1}^{rW}\sum_{y=1}^{rH}(I_{x,y}^{HR}-G\Psi_{G}{\left(I^{LR}{)}_{x,y}\right)}^{2},$$

(10) $$l_{G}=\sum_{n=1}^{N}-logD_{\Psi_{D}}\left(G\Psi_{G}\left(I^{LR}\right)\right).$$

The reduction in PSNR and SSIM metrics has been attributed to the smoothing effect of the reconstructed image through the utilization of the GAN architecture, as reported in the reference [46]. The authors of the study have augmented the all-inclusive loss function of the model by integrating an extra registration loss component, with the objective of enhancing the restoration of high-frequencies [46]. The concept of “Residual Localisation Error” (RLT) is utilized to refer to the anticipated discrepancy between the calculated and actual results of the spatial transformation. The main aim is to reduce the loss of complex details that may occur during the geometric transformation of consecutive frames. The action referred to above is executed with the intention of aiding in the recovery of the original image with an increased level of precision. The diagram provided below illustrates the RLT loss function.

(11) $$RLT=\sum_{i=\pm 1}∥I_{t+i}^{{}^{\prime }LR}-I_{t}^{LR}{∥}^{2}.$$

The aforementioned equation illustrates the outcome of the application of the registration network on the image denoted as $I_{t+i}^{LR}$. The aforementioned process results in the emergence of a novel visual representation denoted as $I_{t+i}^{{}^{\prime }LR}$. The subsequent equation denotes the magnitude of the center of gravity’s length, represented by the variable $I_{G}$:

(12) $$I_{G}=l_{MSE}+10^{-6}I_{G}+\varrho RLT.$$

Based on the outcomes of the experiment, the weight coefficient for RLT, denoted by the variable ϱ, has been determined to be 0.001. Regarding the notion of Wasserstein Generative Adversarial Networks (WGAN), it is possible to exclude the variables $l_{Gen}$ and $I_{D}$, resulting in a modification of the objective function $l_{Gen}$.

(13) $$l_{Gen}=\frac{1}{N}\sum_{n=1}^{N}D_{\Psi_{D}}\left(G\Psi_{G}\left(I^{SR}\right))-D_{\Psi_{D}}\left(I^{HR}\right)\right).$$

### 4.1. Registration of DWI Scans

The efficacy of the motion estimation in the network, which utilizes a multi-scale methodology, has been established in conventional techniques, as indicated by prior research [29]. The process receives input consisting of the target frame, which is identified as $I_{t}^{LR}$, and the adjacent frame, which is denoted as $I_{t-R:t+R}^{LR}$. The image undergoes motion correction via pyramidal registration techniques, which involve the training and utilization of spatial transformation parameters. The registration of a three-image input entails the distinct alignment of a pair of images via the registration layer. The process of optimizing the parameters of the spatial transform network can be accomplished by minimizing the mean-squared error between the compensated scan and the reference one, which is represented by the symbol $\omega_{\delta ,t+1}^{*}$. The utilization of this specific training methodology amplifies the neural net’s capability to conduct motion compensation on the given image set.

(14) $$\omega_{\delta ,t+1}^{*}=\underset{\omega_{\delta ,t+1}}{argmin}∥I_{t}^{LR}-I_{t}^{{}^{\prime }LR}{∥}^{2}.$$

The notation $I_{t}^{{}^{\prime }LR}$ represents the outcome of the registration layer denoted as $I_{t}^{LR}$ that follows the registration procedure.

The sequential arrangement of the images is under observation. The diagram illustrated in Figure 1 represents the configuration of the network layer accountable for executing the registration procedure. The effectiveness of deformable registration representation in classical methods has been demonstrated by various sources, including references [47,48]. These sources implemented a multi-scale design to achieve their results.

The current investigation utilizes an algorithm to derive a spanning tree that exhibits the lowest collective edge costs. The nodes denoted by the symbol i∈P denote discrete entities that could potentially exist as singular pixels or as agglomerations of pixels. Each node is associated with a set of hidden labels that correspond to motion fields, expressed as $w_{i}^{l}=\left\{f_{i}^{l},g_{i}^{l},h_{i}^{l}\right\}$. The energy function utilized for optimization comprises of two distinct components, namely, the data cost denoted by the symbol *S*, and the pair-wise regularization cost denoted by $R(w_{i}^{l},w_{i}^{m})$ for all nodes L that are interconnected with nodes *m*.

(15) $$E\left(w_{i}\right)=\sum_{i\in P}S\left(w_{i}^{l}\right)+\kappa \sum_{l,m\in N}R\left(w_{i}^{l},w_{i}^{m}\right).$$

The aforementioned cost function provides an estimation of the similarity of pixels between two compared images. This term remains unaffected by the displacements exhibited by its adjacent entities. The utilization of the parameter κ serves the purpose of assigning weight and determining the impact of the regularization factor. The first component of the equation denoted as (15) (expressed as $\sum_{i\in P}S\left(w_{i}^{l}\right)$) pertains to the data term, whereas the subsequent component (expressed as $\kappa \sum_{l,m\in N}R\left(w_{i}^{l},w_{i}^{m}\right)$) pertains to the regularization term.

### 4.2. The DWI Images Deblurring Network

**Figure 6:**
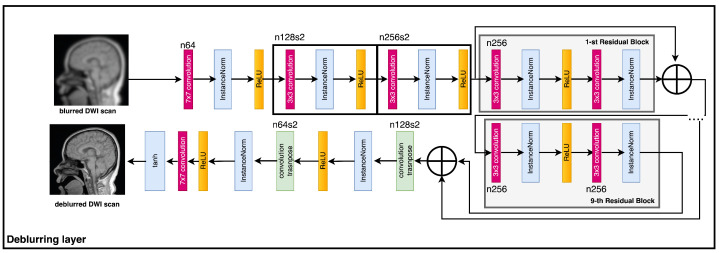
Deblurring net.

The aim of this study is to achieve the retrieval of a well-defined and specific visual depiction, referred to as $I_{S}$, through the utilization of a blurred visual representation, $I_{B}$, as the exclusive input, without any supplementary knowledge regarding the blur kernel. The deblurring procedure involves the utilization of a convolutional neural network that has undergone training, referred to as $G\rho_{G}$ and commonly recognized as the Generator. An estimation is computed for the corresponding image of the saturation current ($I_{S}$) for every value of the base current ($I_{B}$). Moreover, during the learning process, the critic network denoted as $D_{\rho_{D}}$ is incorporated, and both networks undergo adversarial training. The composite loss function is derived by combining content and adversarial losses:

(16) $$L=L_{G-A-N}+\lambda \cdot L_{X},$$

where $L_{G-A-N}$ and $L_{X}$ denote adversarial loss and content loss, respectively. The experimental trials maintained a constant value of 100 for the parameter λ. The present study’s methodology differs from that of Isola et al. [49] as it does not entail the conditioning of the discriminator. The lack of requirement to enforce sanctions for discrepancies between the input and output is the rationale behind this. The notion of adversarial loss constitutes a fundamental element within the domain of machine learning. The aforementioned expression pertains to the utilization of a loss function that is specifically crafted to incentivize the generation of outputs that bear a striking resemblance to those generated by a human or a reference model. The effectiveness of this approach has been proven in various fields, including but not limited to image and speech identification, processing of natural language, and other related domains. Scholars and professionals have discovered that incorporating adversarial loss into machine learning models can improve their accuracy and robustness. This phenomenon leads to improved performance and greater reliability of results (Figure 6).

The formulation for calculating the loss can be defined as follows:

(17) $$L_{G-A-N}=\sum_{n=1}^{N}-D_{\rho_{D}}\left(G\rho_{G}\left(I^{B}\right)\right).$$

The research results outlined in reference [50] indicate that the DeblurGAN model exhibits convergence in the absence of the Generative Adversarial Network (GAN) component during its training phase. Nevertheless, the images produced by the model exhibit features that encompass both fluidity and indistinctness, as well as the concept of content loss. When exclusively optimizing the aforementioned functions, the resulting images may contain ambiguous anomalies. The observed deviations can be attributed to the average potential solution values at the pixel level within the pixel space, as referenced in [24]. The implementation of the L2-loss approach within the perceptual loss function facilitates the computation of the dissimilarity between the convolutional neural network (CNN) feature maps of the produced image and the desired image using a mathematical expression. The aforementioned terminology is articulated as follows:

(18) $$L_{X}=\frac{1}{U_{k,n}B_{k,n}}\sum_{x=1}^{U_{k,n}}\sum_{y=1}^{B_{k,n}}{\left(\emptyset_{k,n}{\left(I^{S}\right)}_{x,y}-\emptyset_{k,n}\left({G_{\rho_{G}}\left(I^{B}\right)}_{x,y}\right)\right)}^{2},$$

where $I^{B}$ is a blurred image defined in the way following way:

(19) $$I^{B}=k\left(M\right)*I^{S}+N$$

The symbol $k\left(M\right)$ is unknown blur kernel determined by motion field *M*.

As per the reference [13], the symbol $\emptyset_{k,n}$ denotes the feature map obtained from the n-th convolution operation, located after activation and before the k-th maxpooling layer, in a pre-existing MRI set network. The feature map dimensions are denoted by the variables $U_{k,n}$ and $B_{k,n}$.

### 4.3. DWI Images Denoising Net

One of the challenges encountered during the denoising procedure for DWI involves the predominance of magnitude images as the principal representation, which are obtained from the real and imaginary components [51]. The Rician distribution is a statistical model that accounts for the manifestation of noise in magnitude images. It is distinguished by a greater degree of intricacy in contrast to conventional forms of additive noise. Several statistical methods have been suggested to model the process of degradation. The level of precision exhibited by the model mentioned above plays a crucial role in determining the final results of the noise reduction process. The utilization of deep learning (DL) presents a potentially efficacious approach to tackle the aforementioned challenge. This phenomenon can be explained by its ability to disregard the underlying physical processes and instead adapt to changes in these processes through the mechanism of sample-based learning.

The objective of mitigating Diffusion Weighted Imaging (DWI) noise is to enhance the quality of the diagnostic image that has been negatively impacted by noise, leading to an improved image. The variable *x* is utilized to represent a DWI image that has been subjected to noise corruption, whereas the variable *y* denotes the DWI image that is devoid of any noise. The matrices represented by the symbols *x* and *y* possess the same dimensions, which are specifically m×n. Additionally, both matrices are composed of elements that are real-valued. The depiction of the relationship between entities can be illustrated as follows:

(20) $$x=\varrho \left(y\right).$$

The mapping function ϱ is utilized to denote the function that represents the presence of noise contamination. The deep learning approach is widely recognized as a technique that operates as a black box, functioning independently of the statistical properties of the noise. Consequently, the optimization of the search for the most suitable approximation of the function $\varrho^{-1}$ has the potential to streamline the procedure of denoising DWI images. The formulation of the denoising process can be expressed as follows:

(21) $$\underset{f}{arg\min}∥\hat{y}-y∥.$$

The function f(x) is used to estimate the dependent variable *y*, while the variable $\hat{y}$ represents the expected outcome. The appropriateness of estimating the inverse of ϱ is commonly attributed to the function f(x).

From a statistical perspective, it is possible to consider *x* and *y* as two separate samples originating from two distinct data distributions. The distributions in question refer to the probability distribution of the noisy image, which is represented as $P_{n}$, and the probability distribution of the noise-free image, which is represented as $P_{synth}$. The denoising procedure involves the utilization of a mapping algorithm to transform a designated distribution into a different distribution. The mapping of samples from the set $P_{n}$ to a distribution $P_{synth}$, which exhibits a significant resemblance to $P_{r}$, is accomplished by the function *f*.

The utilization of Generative Adversarial Networks (GANs) has been widespread across multiple domains, such as image super-resolution, image modality transformation, and image synthesis. The domains mentioned above have been thoroughly investigated, as evidenced by the extant literature [49,52,53]. The discriminative model was created to differentiate between the origin of a particular sample, determining whether it is derived from the generative model’s distribution or the authentic data’s distribution. The primary aim of the generative model is to generate a new instance that closely resembles the authentic data distribution, with the input instance acting as a reference point.

The expression presented denotes the loss function employed for the denoising layer:

(22) $$L_{WGAN}\left(D\right)=-E_{y∼P_{r}}\left[D\left(y\right)\right]+E_{x∼P_{n}}\left[\log D\left(y\right)\right]+E_{x∼P_{n}}\left[D\left(G\left(x\right)\right)\right]+\psi E_{\hat{x}∼P_{\hat{x}}}\left[{\left(∥\nabla_{\hat{x}}D\left(\hat{x}\right){∥}_{2}-1\right)}^{2}\right].$$

The ultimate element of the equation denotes a gradient penalty term, where the penalty parameter is denoted by the symbol ψ. The probability distribution denoted as $P_{\hat{x}}$ is derived through the uniform sampling on straight lines that link two of points sampled from the actual data distribution, $P_{r}$, and the generator distribution, $P_{synth}$. The following is the presentation of the loss function formulation for the generator G.

(23) $$L_{WGAN}\left(G\right)=E_{x∼P_{r}}\left[\log D\left(y\right)\right]+E_{x∼P_{n}}\left[\log\left(1-D\left(G\left(x\right)\right)\right)\right].$$

The Mean Squared Error (MSE) loss function is a frequently utilized approach in tasks that involve modifications at the pixel level. The primary objective is to minimize the disparities between the genuine depiction and the generated representation on a pixel-by-pixel basis. The aforementioned computation can be derived utilizing the subsequent methodology:

(24) $$L_{MSE}=\frac{1}{opr}||G\left(x\right)-{y||}^{2}.$$

The dimensions of the image are represented by the variables *o*, *p*, and *r*. Recent research has indicated that the Mean Squared Error (MSE) loss function can lead to a noteworthy improvement in the peak signal-to-noise ratio. The envisaged result of this methodology is a decrease in precision, specifically in relation to frequently recurring particulars, which could significantly affect clinical diagnosis [52].

The proposed loss function can effectively tackle the issue by integrating a perceptual loss, as mentioned in references [35,51,54]. Utilizing a pre-existing neural network constitutes a feasible strategy for distinguishing distinctive characteristics between genuine and synthetically produced images. Perceptual similarity refers to the extent of dissimilarity between the characteristics of a genuine image and a generated image. The subsequent explanation pertains to the perceptual loss function.

(25) $$L_{perceptual}=\frac{1}{ghi}||\omega \left(G(x)\right)-\omega \left(y\right){\left|\right|}_{F}^{2}.$$

The variable ω is employed as a tool for extracting features, while the dimensions of the feature map are denoted by *g*, *h*, and *i*. The present study employs the pre-existing VGG-19 neural network [55] to extract image features. The VGG-19 convolutional neural network is composed of nineteen layers, with the initial sixteen layers being convolutional and the final three layers being fully connected. The process of extracting features is limited solely to the first sixteen layers. The execution of the perceptual loss based on the VGG network is carried out in a sequential manner as follows:

(26) $$L_{VGG}=\frac{1}{abc}||VGG\left(G(x)\right)-VGG\left(y\right){\left|\right|}_{F}^{2}.$$

**Figure 7:**
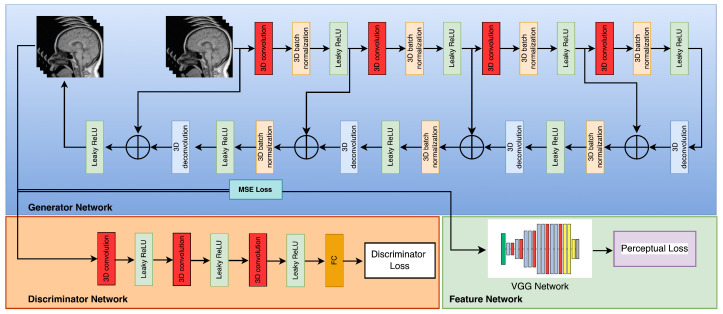
Denoising net.

The generator denoted by G is associated with a composite loss function that is weighted and encompasses the Mean Squared Error (MSE) loss, VGG loss, and discriminator loss. Figure 7 illustrates the schematic representation of the discriminator network, which is referred to as D. The model proposed in this study comprises three convolutional layers, with each layer utilizing filters of different magnitudes, specifically 32, 64, and 128. The configuration of the convolution layers has utilized a uniform kernel size of 3 × 3 × 3. The highest tier is composed of a completely interconnected layer that exclusively generates a singular result. The VGG-19 network, which has been pre-trained, is utilized for the purpose of feature extraction. For further elucidation, those who express interest may refer to the primary source material as recorded in reference [55]. Pan and Yang (2019) have shown that the utilization of transfer learning eliminates the need for retraining the neural network for the targeted MRI images [56]. The aforementioned discovery implies that transfer learning may constitute a viable strategy for enhancing the efficacy of neural networks in the context of medical imaging implementations.

(27) $$L_{RED-WGAN}=\delta_{1}L_{MSE}+\delta_{2}L_{VGG}+\delta_{3}L_{WGAN}\left(G\right).$$

The diagram illustrating the structure of the RED-WGAN model proposed can be found in Figure 7. The system is composed of three discrete components, namely, a generator network labeled as G, a discriminator network labeled as D, and a VGG network employed as a feature extractor. The convolutional and deconvolutional layer pairs are connected through short connections in a corresponding manner. With the exception of the last layer, each layer performs a series of operations that include three-dimensional convolution, activation through Leaky-ReLU, and normalization through batch processing. The ultimate layer exclusively executes a 3D convolution operation and applies a Leaky Rectified Linear Unit activation function. The current investigation utilizes a 3 × 3 × 3 kernel setup for all kernels and implements a filter sequence comprising of 32-64-128-256-128-64-32, and 1.

## 5. Results

**Figure 8:**
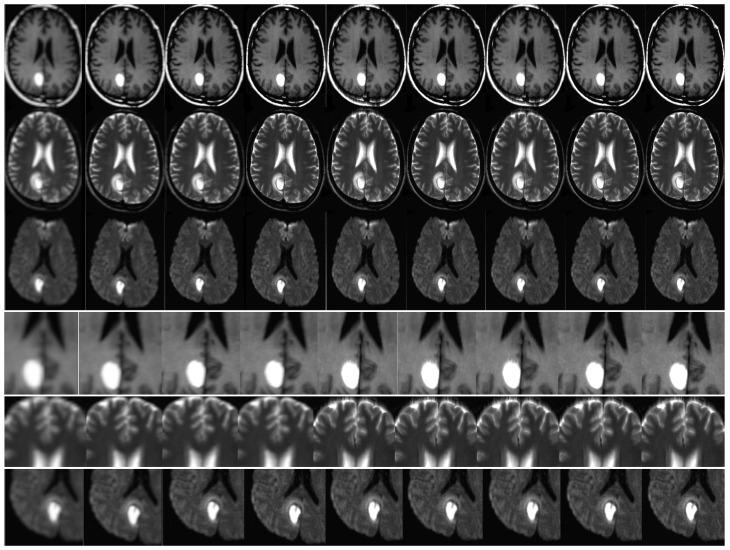
This study assessed the efficacy of diverse super-resolution image reconstruction techniques for Magnetic Resonance Imaging (MRI) T1, T2, and Diffusion Weighted Imaging (DWI) images, using clinical illustrations. The findings of this assessment indicated the existence of a hyperintense hematoma located in the right parietal lobe. The methodology utilized in the investigation comprises of LR input, B-spline Cubic interpolation, Non-rigid multi-modal medical image registration by combining L-BFGS-B with cat swarm optimization (Yang’s N-R-M-3D) [57], Enhanced Deep Residual Networks for Single Image Super-Resolution (Lim’s E-D-R-N-S-I-S-R [58]), image super-resolution using very deep residual channel attention networks (Zhang’s R-D-N-I-S-R [59]), Residual dense network for image super-resolution (RCAN SRR [60]), MRI Super-Resolution using GAN and DWT [43], Brain MRI SRR using 3DGAN [44] and the algorithm suggested by the author. The techniques were organized in a horizontal sequence from the leftmost to the rightmost position. The aforementioned techniques are supported by references [4,61], and [58], as cited in the study. Zoomed-in views are shown in the last three rows.

**Figure 9:**
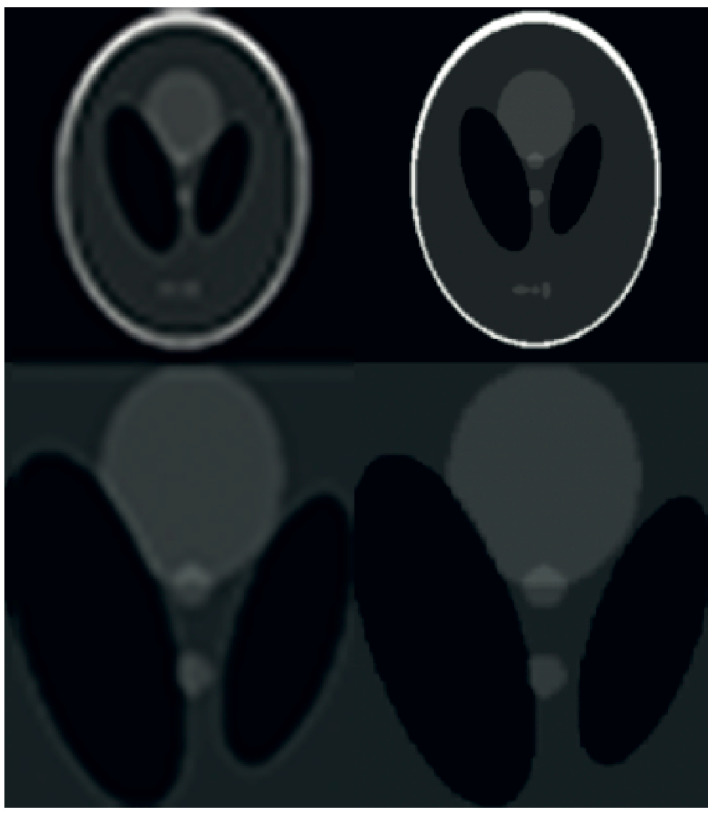
A comparison of results obtained from the Shepp–Logan phantom. The reconstructed output of the PROPELLER sampling pattern is presented on the left, while the outcome of the proposed algorithm with improved resolution is displayed on the right. The bottom row reveals intricate visuals.

The effectiveness of the algorithm was assessed through the analysis of in vivo patient cases and phantom data. The MATLAB software was utilized for conducting all trials and investigations. The objective of the assessments was to assess the effectiveness of super-resolution image reconstruction through the utilization of compressed input. An additional aim was to assess the efficacy of the MR sampling technique. This study involved the analysis of both in vivo and phantom data. The simulation results are illustrated in Figure 8 and Figure 9. It is noteworthy that the utilization of Compressed Sensing in conjunction with its conjugate symmetry and partial Fourier technique expedites the process of data acquisition in comparison to alternative unaltered k-space sampling patterns. The analysis of human data involved the processing of Siemens Prisma scanner files, which had a measurement of 3.0 terabytes. In order to replicate the performance of a multi-channel head coil across a range of in-plane resolutions, diffusion directions, and slice thicknesses, fully sampled k-q spaces have undergone decimation. The denoising problem is defined within the framework of Maximum A Posteriori (MAP) estimation by the proposed method. The algorithm for noise reduction under consideration yields an average value of approximately 2.1 dB, as determined by the tests conducted. Phantom-based experiments were conducted on high-quality DW multi-shot EPI sequences. The simulation of off-resonance effects was conducted utilizing fugue. The study employed D-BRAIN phantom models, which are diffusion MRI brain data models that are anatomically precise. The training of the model was conducted on an NVIDIA DGX machine equipped with GPU A100, utilizing the resources of Google Colab Pro. The generator undergoes training using a pair of LR images, which are stacked and have dimensions of 60 × 60 × 2. The objective is to generate a single HR image with dimensions of 240 × 240. The generator employs a tripartite loss function comprising of content loss, perceptual loss, and adversarial loss. The discriminator is subjected to both the SR and HR images obtained from MRI. The binary classifier known as the discriminator employs binary cross-entropy as its optimization technique. The utilized optimizer is Adam, which is applied to both the generator and discriminator. The learning rate is $10^{-5}$ (Table 1, Table 2, Table 3, Table 4 and Table 5).

The present study incorporates the technique of compressed-sensing with super-resolution image reconstruction for application in Magnetic Resonance Imaging scanners. The utilization of phantom data has been employed to expose the inverse challenges associated with compressed sensing in the context of magnetic resonance imaging (MRI). The utilization of sparse projections has been observed in the retrieval of Shepp–Logan phantom data, as depicted in Figure 8. Twenty-five and twelve radial lines in the frequency domain are capable of reconstruction. The data reconstruction process employed by the author involves utilizing a subset of sixty projections within a ninety-degree aperture to reconstruct data from limited-angle projections. The method employed for the creation of all Shepp–Logan phantom images was this. The input frame was degraded by a nonrigid transformation. The application of rotation and subpixel shift factors was carried out in the following manner. Local affine simulations were utilized to model motion artifacts of internal organ tissue in order to demonstrate blurring. The low-resolution photos were subjected to a Gaussian blur kernel resulting in blurring. Subsampling using a predetermined ratio will ensue. The addition of white Gaussian noise occurred subsequently. The noise level of the test photos was measured to be twenty-five standard deviations. The recording of deformable medical images presents a challenge. The utilization of Markov random field (MRF) optimization in a registration method based on structural representation reduces the dimensionality of transformation parameters and imposes constraints on energy function values in the image region associated with non-rigid deformation.

This study employs various descriptors, namely, the hybrid L-BFGS-B and cat swarm optimisation (HLCSO), sum of squared differences on entropy images, MIND (Modality independent neighbourhood descriptor), and self-similarity context (SSC).

The assessment of motion estimating algorithms has employed Target Registration Error (TRE) as a metric.

(28) $$TRE=\frac{1}{N}\sum_{i=1}^{N}\sqrt{{\left(T_{L_{x}}-T_{D_{x}}\right)}^{2}+{\left(T_{L_{y}}-T_{D_{y}}\right)}^{2}+{\left(T_{L_{z}}-T_{D_{z}}\right)}^{2}}.$$

The variable $T_{L}$ denotes the forced deformation, which is obtained through a linear combination of radial basis functions and serves as the reference ground truth. On the other hand, the variable $T_{D}$ represents the estimated deformation parameters that are obtained through the motion estimation techniques presented in Table 6. Furthermore, the variable *N* denotes the quantity of landmarks that have been manually marked according to the recommendations of medical professionals. The study aimed to evaluate the effectiveness of the the WGAN-based deformable motion registration algorithm by comparing it to several image registration techniques in order to identify the most successful approach. The statistical analysis was performed on each of the registration algorithms listed by utilizing the mean and standard deviation. (std). Table 6 presents the computed values of Type I error rate (TRE) and probability (P) for the t-test conducted at a significance level of 0.05. The statistical analysis reveals that the the author’s method exhibits a significant superiority over its competitors, as evidenced by all p values being less than 0.002. The registration of input images using the “deeds” method resulted in mean and standard deviation values of 1.4 voxels and 0.2 voxels, respectively, for the TRE values. These values are demonstrated to be more favorable compared to other algorithms, as presented in Table 6.

## 6. Discussion

The limitations of medical imaging modalities, such as low spatial resolution, weak contrast, visual noise scattering, and blurring caused by the complexity of internal body tissues, can have a substantial impact on the accuracy of medical diagnosis. The text outlines a number of improvements that are purported to be advantageous in augmenting the quality of images while simultaneously minimizing scanning durations. Despite the presence of motion distortions, the algorithm that has been suggested effectively mitigates artifacts that are caused by data that has been highly undersampled. The algorithm presented in this study integrates an effective highly sparse acquisition strategy, namely, partial Fourier and Poisson Disc sampling, with super-resolution reconstruction to yield superior outcomes. The outcome is an improvement in the quality of dMR images and a decrease in time complexity. Furthermore, the augmentation of sampling for high-frequency components results in an improvement of edge depiction. The aforementioned methodology has the potential to be executed on dMR scanners without necessitating any alterations to the hardware. Therefore, it is apparent that the algorithm that has been implemented has the capability to generate images that are improved and more accurate. This study investigates the viability of a newly proposed DW-MRI algorithm, which aims to enable rapid and effective high-resolution diffusion imaging. The algorithm in question, known as RoSA, operates by selecting a single compressively sensed rotating blade of a PROPELLER in each diffusion direction. Moreover, the utilization of the resemblances among contiguous DW images constitutes a pivotal facet of the method’s execution. The short-axis EPI readout model has retained the advantages of short-axis EPI with regards to distortion reduction and significant improvement in resolution along the phase-encoding direction as a result of T2 decay.

Improving spatial resolution yields clear advantages for both clinical diagnosis and fundamental research. Early detection of soft tissue abnormalities is possible. Furthermore, as a result of the reduction in movement aberrations, the algorithm has the capacity to augment the perceptibility of white matter tractography, as well as the trajectory of nerve fibers in close proximity to neoplasms. In comparison to T1/T2-weighted imaging, which generally offers isotropic resolutions of 1.0 mm or more for complete brain coverage, diffusion MRI is constrained by either excessively large voxel sizes or insufficient coverage. The proposed algorithm offers a major advantage in terms of enhancing image resolution and reducing examination time, in comparison to existing solutions. The aforementioned outcome is attained by means of amalgamating super-resolution image reconstruction, accurate motion estimation, and capitalizing on the similarity in the sequence of diffusion weighted (DW) images of neighboring diffusion directions. The efficacy of the algorithm was evaluated through a range of studies carried out under varied circumstances, wherein the quantity of PROPELLER blades, overall diffusion directions, and window dimensions were altered within the range of 5 to 40. In addition, the existence of off-resonance artifact is commonly acknowledged as a significant obstacle to EPI-based acquisition protocols. The implementation of improved sampling techniques has led to the reduction of aberrations and the mitigation of motion-induced distortions.

The enhanced precision of tissue demarcation heightens the probability of arriving at a precise clinical diagnosis, for reasons that are self-evident. This mode entails certain financial considerations that necessitate attention. Hence, it is yet to be ascertained whether the increased procurement expenses associated with DW-MRI would be rationalized in the foreseeable future. The objective of this study was to showcase the capabilities of integrating various methodologies to enhance DW-MRI images, while simultaneously preserving a swift acquisition rate. As per anticipated outcomes, the algorithm put forth has the potential to enhance image resolution without necessitating any alterations to the hardware. Moreover, this approach has the potential to mitigate motion distortions while compromising on resolution. Preliminary trial data can serve as a valuable source of background information that may aid in the reduction of examination duration. The utilization of motion estimation algorithm can significantly reduce the presence of image artifacts in diagnostic imaging, thus enhancing the probability of an accurate diagnosis.

Please refer to Figure 7 and Figure 8 for empirical support of the enhancement in both image resolution and quality. The improved resolution and legibility of the procedures utilized for potentially malignant or premalignant lesions have resulted in an increased capacity to detect them. The method’s anticipated efficacy is largely attributed to its potential advantages, such as achieving a heightened contrast while preserving a superior level of resolution. The present study investigates the integration of magnetic resonance (MR) with super-resolution and compressed-sensing techniques. The enhancements that were obtained have led to notable improvements in sharpness, edge interpretation, and contrast. The achievements have been verified by PSNR as well. The impact of CS quality ratios on PSNR values is significant, as demonstrated by the Table 1, Table 2, Table 3, Table 4 and Table 5.

The average peak signal-to-noise ratio (PSNR) is computed by utilizing various method outputs and ground truth images. The simulation was conducted on one hundred occasions. To ensure the statistical reliability of quality measurements, the PSNR calculation procedure was replicated for every simulation case and subsequently averaged. It is noteworthy that the most favorable outcomes were achieved utilizing a compression ratio of 50%. This implies that a further reduction in the overall quantity of input samples leads to a slight improvement in the peak signal-to-noise ratio (PSNR), as evidenced by the data presented in Table 1, Table 2, Table 3, Table 4 and Table 5. The reduction in exam duration is directly proportional to this particular value. Furthermore, this approach has the potential to mitigate motion distortions in addition to compromising on resolution. The approach centers on expediting the convergence of algorithms, incorporating image prior, and recognizing blur kernels. Preliminary trial data can serve as valuable background information to expedite the duration of examinations. Notwithstanding the capability of the motion estimation algorithm to significantly mitigate diagnostic image artifacts, thereby enhancing the probability of an accurate diagnosis; it is incapable of completely eliminating all artifacts. Figure 8 and Figure 9 demonstrate an enhancement in the resolution and caliber of the outcomes acquired. The results mentioned above are provisional and susceptible to modification. The algorithm’s advantages were demonstrated through a qualitative evaluation of the neuroimages of twenty patients. The study involved the collection of DW-MRI data from twenty patients with oncological conditions, using a single scanner. The calculation of the mean square error (MSE) was performed for each instance of reconstruction in order to obtain statistically significant measurements. The study employed the signed rank test to examine the null hypothesis that the central tendency of the difference across different sparsity rates was equivalent to zero. The statistical analyses were conducted using the R Project for Statistical Computing. A statistical analysis was performed utilizing a t-test for independent groups to compare the mean values of peak signal-to-noise ratio (PSNR) between two distinct groups. Additionally, a paired t-test was conducted to analyze paired data. The algorithm’s robustness has been confirmed by the probability, which was determined through a significance test and Student’s t-test conducted on the PSNR. The outcomes are exhibited in Table 3, Table 4 and Table 5. The statistical significance of the results was high, as indicated by the obtained p-values.

The study conducted a comparison between the proposed algorithm’s robustness and other existing image resolution enhancement algorithms, namely, bicubic spline interpolation, Yang’s N-R-M-3D [57] (3DKR) as cited in reference [4], and IBP that utilizes optical flow-based motion estimation as its fundamental technique. The results of the comparison are presented inTable 1, Table 2, Table 3, Table 4 and Table 5. The results are visually represented through the use of graphical illustrations, as evidenced by Figure 8. The obtained outcomes have been contrasted with four advanced super-resolution image reconstruction techniques, namely, sightly modified the MRI super-resolution using GAN and DWT [43] and the brain MRI super-resolution using 3D GANs [44] Yang’s N-R-M-3D [57], Lim’s E-D-R-N-S-I-S-R [58], RCAN SRR [60], and Zhang’s R-D-N-I-S-R [59]. The Lim’s E-D-R-N-S-I-S-R [58], RCAN SRR [60], z z Zhang’s R-D-N-I-S-R [59] methods employ the L1-loss function for network training purposes.

Compressed sensing (CS) theory provides a feasible approach to reconstructing signals with sparsity by projecting them onto a linear subspace of low dimensionality. This methodology exhibits significant potential owing to the theoretical assurances it offers. The methodology employed in this study involves the integration of Generative Adversarial Networks (GANs) in the image domain, along with k-space corrections. Generative Adversarial Networks (GANs) are accountable for encoding prior knowledge that is specific to images. Hence, it is imperative to establish a spurious linkage between the input and output strata of Generative Adversarial Networks (GANs). Possible areas for future research include enhancing reconstruction algorithms and refining raw data sampling schemes.

## Figures and Tables

**Table 1:** The performance of the SRR algorithm under consideration.

Sparsity Rate * [%]	N	PSNR	SD	t	p
20	100	19.22	0.02	0.021	1.231
40	100	24.43	0.02	0.995	0.098
60	100	31.21	0.03	−0.631	0.431
80	100	33.77	0.04	0.001	0.923
100	100	36.19	0.03	−1.232	0.069

* The term “sparsity rate” pertains to the proportion of input samples that remain after a comparison with fully sampled scans. For instance, a sparsity rate of 60 indicates that 40% of the samples from a fully sampled scan have been eliminated.

**Table 2:** The statistical parameters of the model’s PSNR metrics were analyzed on the aforementioned Figure 8.

Reconstruction Algorithm	N	PSNR	SD	t	p
No SRR. MC-not-applied	100	26.09	0.01	−1.291	0.131
B spline. MC-not-applied	100	28.01	0.01	−0.192	0.621
Yang’s N-R-M-3D [57]	100	31.20	0.01	−0.592	0.382
Lim’s E-D-R-N-S-I-S-R [58]	100	30.21	0.01	−0.292	0.312
Zhang’s R-D-N-I-S-R [59]	100	29.91	0.01	−0.391	0.391
RCAN SRR [60]	100	31.23	0.01	−0.311	0.231
MRI Super-Resolution using GAN and DWT [43]	100	31.78	0.01	−0.232	0.159
Brain MRI SRR using 3DGAN [44]	100	32.66	0.01	−0.114	0.162
The author’s method	100	34.23	0.00	−1.001	0.012

**Table 3:** This study evaluates the efficacy of various reconstruction algorithms for in vivo brain imaging, specifically in the context of MC-motion correction. Please refer to Figure 8 for the relevant image.

HR Image Reconstruction Method	N	PSNR	SD	t	p
no SRR/MC-not-applied	100	27.21	0.01	−1.144	0.254
no SRR/MC-applied	100	29.99	0.01	0.323	0.665
B-spline/MC-not-applied	100	30.77	0.01	−0.312	0.555
the author’s method/MC-applied	100	35.22	0.01	0.031	1.102

**Table 4:** The statistical parameters pertaining to the PSNR metrics of the model are presented in relation to Figure 9.

Sparsity Rate [%]	N	PSNR	SD	t(99)	p
20	100	29.77	0.01	−1.001	0.192
40	100	32.22	0.03	−1.032	0.111
60	100	35.66	0.01	−1.041	0.132
80	100	37.02	0.01	−1.046	0.211
100	100	37.11	0.01	−1.022	0.100

**Table 5:** The present research assesses the effectiveness of diverse reconstruction algorithms for in-vivo brain imaging, encompassing MC-motion correction and HR-upscaling through the utilization of the presented algorithm. Kindly consult Figure 4 for the corresponding cerebral image.

The Algorithm for Sampling and Reconstructing k-Space	N	PSNR	SD	t(99)	p
P-R-O-P-E-L-L-E-R	100	21.96	0.03	−1.357	0.178
The author’s sampling scheme	100	34.49	0.04	1.286	0.202

**Table 6:** The statistical performance of numerous registration algorithms vs. the applied procedure.

Motion Compensation Procedure	TRE [Voxels]		
	Mean	Std	*p*-Value
not-applied	4.7	2.2	
Wachinger’s [62]	2.2	0.9	<0.002
Yang’s et al. [57]	2.2	0.3	<0.002
MIND [63]	1.8	0.1	<0.002
WGAN deformable MC-the author’s method	1.4	0.2	<0.001

## Data Availability

Data is unavailable due to privacy or ethical restrictions.
